# Animal and Vegetable Physiology

**Published:** 1854-12

**Authors:** 


Animal and Vegetable Physiology.—Dr. Salisbury, of Albany, has communicated to the American Scientific Association, some experiments on plants, which illustrate the analogy existing between animal and vegetable physiology. He extracted the poison of a dead rattlesnake, a small portion of which he inserted in the plants by moistening with it the blade of a knife, with which he wounded a lilac, a horse-chestnut, a corn plant, and a sunflower. In sixty hours after the infliction of the wound, they began to manifest symptoms of poisoning, and in a few days all the leaves above the wound were dead. In about fifteen days they manifested convalescence, and nearly all recovered from the injury.

